# Molecular Adjustment to a Social Niche: Brain Transcriptomes Reveal Divergent Influence of Social Environment on the Two Queen Morphs of the Ant *Temnothorax rugatulus*

**DOI:** 10.1111/mec.17649

**Published:** 2025-01-07

**Authors:** Marah Stoldt, Matteo Antoine Negroni, Barbara Feldmeyer, Susanne Foitzik

**Affiliations:** ^1^ Institute of Organismic and Molecular Evolution Johannes Gutenberg University Mainz Germany; ^2^ Department of Biology University of Fribourg Fribourg Switzerland; ^3^ Senckenberg Biodiversity and Climate Research Center (SBiK‐F) Molecular Ecology Frankfurt Germany

**Keywords:** alternative reproductive strategies, gene expression, polygyny, social insects

## Abstract

Social insects form complex societies with division of labour between different female castes. In most species, a single queen heads the colony; in others, several queens share the task of reproduction. These different social organisations are often associated with distinct queen morphologies and life‐history strategies and occur in different environments. In the ant *Temnothorax rugatulus*, queens are dimorphic. Macrogynes and microgynes reside in mono‐ and polygynous colonies and at lower and higher elevations, respectively. We analysed plastic changes in brain transcriptomes in response to the social environment in these queen morphs and their workers. We manipulated the number of queens over 4 months to investigate whether transcriptional activity is influenced by queen morph, social environment or their interaction. Changes in gene expression in the queens' brains in response to our manipulations were largely influenced by the interaction between social environment and queen morph, rather than independently by these factors. Macrogynes and microgynes thus adjust differently to their social environment. Similarly, worker transcriptomes were influenced by an interaction between behavioural type, that is, nurses or foragers, and queen morph. Nurses differentially regulated genes related to nutrition depending on queen morph, suggesting a link between social environment and metabolic dynamics in ant colonies. Overall, our study sheds light on how the social environment influences the molecular physiology of social insects. Furthermore, we demonstrate that in this ant with two queen morphs, worker physiology depends on queen morph and their role in the colony.

## 1 Introduction

Polymorphisms, that is, the production of multiple morphological phenotypes within the same species and sex, are a common phenomenon in insects, especially in social insects. Alternative adult phenotypes are often plastic and regulated via food quality and quantity or temperature during larval development. In other cases, genetics can determine or at least influence morphology (Wheeler, Buck, and Evans 2006; Schwander et al. 2010; Berens, Hunt, and Toth 2015). In ants, the same genomes can give rise to a variety of adult phenotypes varying in morphology, physiology, behaviour and life histories. The reproductive division of labor between female castes represents an excellent example of how divergent these phenotypes can be, as ant queens and workers often differ several folds in body size, fecundity and lifespan (Hölldobler and Wilson 1990). Other traits remain plastic throughout an ant's lifetime, as exemplified by the behavioural division of labor among workers, which results in task specialisation often without morphological differentiation (Kohlmeier, Feldmeyer, and Foitzik 2018).

The ancestral queen phenotype is that of a well‐provisioned large queen that, following nuptial and dispersal flights, will found a new colony on her own (Boulay et al. 2014). However, the trade‐off between the costs and benefits of dispersal has probably led to the evolution of alternative queen morphs that vary in their dispersal capabilities (Rüppell and Heinze 1999; Helms 2018; Hakala, Seppä, and Helanterä 2019). The second queen morph that frequently arose during ant evolution are smaller queens that mate within or close to the mother nest, before seeking re‐adoption to reproduce side by side with other queens in polygynous nests. These two alternative forms of reproduction are therefore typically associated with social colony organisation, with the large, independently founding queens residing in monogynous societies and the dependent, smaller queens living in polygynous colonies (Keller and Ross 1993). Besides different dispersal strategies, the two reproductive queen types differ in several life‐history traits. Theory and comparative evidence predict monogynous queens to exhibit longer lifespans, as the death of the queen also means the death of the colony (Nonacs 1988; Keller and Genoud 1997). In polygynous colonies, a dead queen can easily be replaced by a young sister queen. Moreover, single queens of polygynous colonies were found to be less fecund as they share reproductive output among one another (see also Vargo and Fletcher 1989; Schrempf, Cremer, and Heinze 2011; Clark and Fewell 2014). Examples of alternative reproductive strategies within the same species include the ants *Solenopsis geminata, Formica selysi* and several *Myrmica* species (McInnes and Tschinkel 1995; Sundström 1995; DeHeer and Tschinkel 1998; Howard 2006; Rosset and Chapuisat 2007). In these species, the size distribution of queens is bimodal, with almost no occurrence of queens of intermediate sizes. Large queens are often referred to as macrogynes, whereas smaller queens are called microgynes. In addition to differences in longevity and fecundity between the two morphs, evidence suggests that in the fire ant *Solenopsis invicta*, workers play a crucial role in discriminating between the two queen morphs. The social environment also affects worker traits such as body size and lifespan (Calabi and Porter 1989; Keller and Ross 1998; Goodisman et al. 1999). In *S. invicta, F. selysi* and several other, not as well‐studied ants, including *Cataglyphis*, *Leptothorax* and *Pogonomyrmex* species, long non‐recombining elements, so‐called ‘social chromosomes’ determine queen morph and dispersal strategy (Wang et al. 2013; Purcell et al. 2014; Brelsford et al. 2020; Yan et al. 2020; Chapuisat 2023). Although queen number and morphology are often closely linked in the above systems, the co‐existence of multiple queens in the same colony has evolved many times from monogynous ancestors in the ants and is often not associated with queen dimorphism (Hughes et al. 2008; Boulay et al. 2014).

The genetic or environmental bases for alternative queen morphs have been studied in several species. Yet, it remains unclear how the social environment affects the molecular physiology of adult queens and their workers. This question is addressed in this study. As cues on the social environment are perceived via the sensory system and processed in the brain (Jernigan and Uy 2023), they may impact gene expression in the central nervous systems of queens and workers. For example, in queens of *S. invicta* (Manfredini et al. 2013, 2022), a primary molecular response to social organisation was detected in the brain. Influence of social environment and of social isolation on transcriptional activity in the brain of workers was also found in *Leptothorax* and *Temnothorax* ants (Scharf et al. 2021; Stoldt et al. 2023). We focus on the ant *Temnothorax rugatulus*, a small facultatively polygynous species from Western North America. Queens of this species show a clear bimodal distribution of body size. Queen morph is associated with queen number, with macrogynes typically residing in monogynous colonies and worker‐sized microgynes generally found in polygynous societies (Rüppell, Heinze, and Hölldobler 1998; Choppin et al. 2021; Negroni et al. 2021). Mixed colonies with queens of both morphs are occasionally found. An experimental quantitative trait analysis indicated heritability of queen morph but also identified multiple other factors, such as colony size and composition, affecting queen size (Rüppell, Heinze, and Hölldobler 2001). Indeed, body size and social organisation are interrelated in complex ways, as in the two queen morphs, body size is inversely linked to queen number. Moreover, the size of workers is also related to queen morph and number (Choppin et al. 2021). The microgynes occur mainly at high elevations, indicating they might cope better with less favourable environmental conditions. Additionally, these queens appear to be as fecund and long‐lived as macrogynes, possibly due to more frequent feeding by workers and a higher metabolic rate (Negroni et al. 2021). Regardless of queen morph, the presence of multiple queens in the colony decreases the egg‐laying rate of each queen. These earlier results suggest that both queen number and morph influence the physiology of these ants, but the extent to which these factors interact and affect workers remains unclear.

In this study, we experimentally disentangled the effect of queen morph and social organisation by using a full‐factorial design. We expected macrogynes to be well adapted to the situation of being the only queen in a colony, whereas for microgynes, this could be an unusual and potentially stressful social environment. Conversely, we predicted that macrogynes might respond to the lower level of care potentially provided in polygynous societies compared to microgynes, for whom polygyny is the typical social organisation (Negroni et al. 2021). We focus here on the transcriptional activity in the brain, as the central nervous system is where information about the social environment is perceived, processed and can lead to physiological or behavioural responses (Manfredini et al. 2022; Jernigan and Uy 2023). There is evidence that behavioural dynamics between workers and queens depend on queen morph, as trophallactic interactions with microgynes are much more frequent (Negroni et al. 2021). In addition to changes in the expression of behavioural genes, we were interested in whether the queens responded to their social environment by altering the expression of genes associated with stress, fertility or lifespan functions. We also investigated the influence of queen form and behavioural task on the brain transcriptome of workers to determine whether *T. rugatulus* workers from macrogynous colonies differ in brain activity and potentially behaviour from those raised and living in microgynous societies.

## 2 Material and Methods

### 2.1 The Effect of Queen Morph on Brain Gene Expression

#### 2.1.1 Ant Collection, Maintenance and Experimental Manipulation

*T. rugatulus* colonies were collected in rock crevices and under stones in oak‐pine forests of the Chiricahua Mountains, Arizona, USA, in August 2015 (coordinates and additional information: Table S1). Collection permits for the Coronado National Forest were obtained through the Southwestern Research Station of the Museum of Natural History in Portal, Arizona. In the laboratory, ants were kept at 22°C with a 12:12 light:dark cycle and fed crickets and honey twice weekly, with water provided ad libitum.

Queens were classified into micro‐ or macrogyne based on the body size index (Rüppell, Heinze, and Hölldobler 1998; Choppin et al. 2021; Negroni et al. 2021) a measure that is closely linked to their dry weight. A total of 91 polygynous colonies (average colony size: 217.71 ± 169.33 workers), 44 with exclusively macrogyne queens and 47 with only microgyne queens, were selected for further experiments (Table S2). To experimentally vary social structure, specifically queen number, we split 86 of the 91 colonies. Each experimental colony received 50 workers with a similar proportion of each behavioural type (18 nurses, 2 guards, 4 foragers and 26 other in‐nest workers for each experimental colony fragment), as well as 12 larvae, while all eggs were removed. Experimental colonies assigned to be monogynous contained one queen of the respective queen morph, whereas those assigned to be polygynous contained two queens of the respective queen morph. Overall, colonies remained under these experimental conditions for over 4 months, allowing queens and workers to adjust to the experimental conditions, that is, colony size and queen number. During the course of the experiment, colony composition stayed consistent across treatments. Neither the number of workers nor queen number in the source colonies differed between the macrogyne and microgyne colonies (Mann–Whitney U‐test: workers: *z* value −0.15, *p* value 0.88; queens: *z* value 1.23, *p* value 0.22), and the same holds true for colonies from the mono‐ and polygynous treatment (Mann–Whitney‐U workers: *z* value −0.46, *p* value 0.65; queens: *z* value −0.09, *p* value 0.92). At the end of the experiment, the head of one queen from six colonies was decapitated and flash‐frozen using liquid nitrogen and stored at −80°C until dissection. The same was done for one nurse and one forager for each of the polygynous colonies.

### 2.2 RNA Extraction, Sequencing and Gene Expression Analyses

Brains were dissected into 50 μL of Trizol and RNA was extracted using the RNeasy mini extraction kit (Qiagen) following the standard protocol. Samples were sent to Beijing Genomics Institute (BGI) Hongkong for sequencing on an Illumina HiSeq 4000, resulting in 20–100 million 100‐bp‐long paired‐end reads per sample. The sequencing failed for one macro‐polygynous queen. Sequences were quality‐ and adapter‐trimmed using Trimmomatic Version 0.38 (Bolger, Lohse, and Usadel 2014) with non‐default parameters HEADCROP:11, TRAILING:3 and SLIDINGWINDOW:4:15, and the quality was assessed using FastQC Version 0.11.8 (Andrews 2015).

**FIGURE 1:**
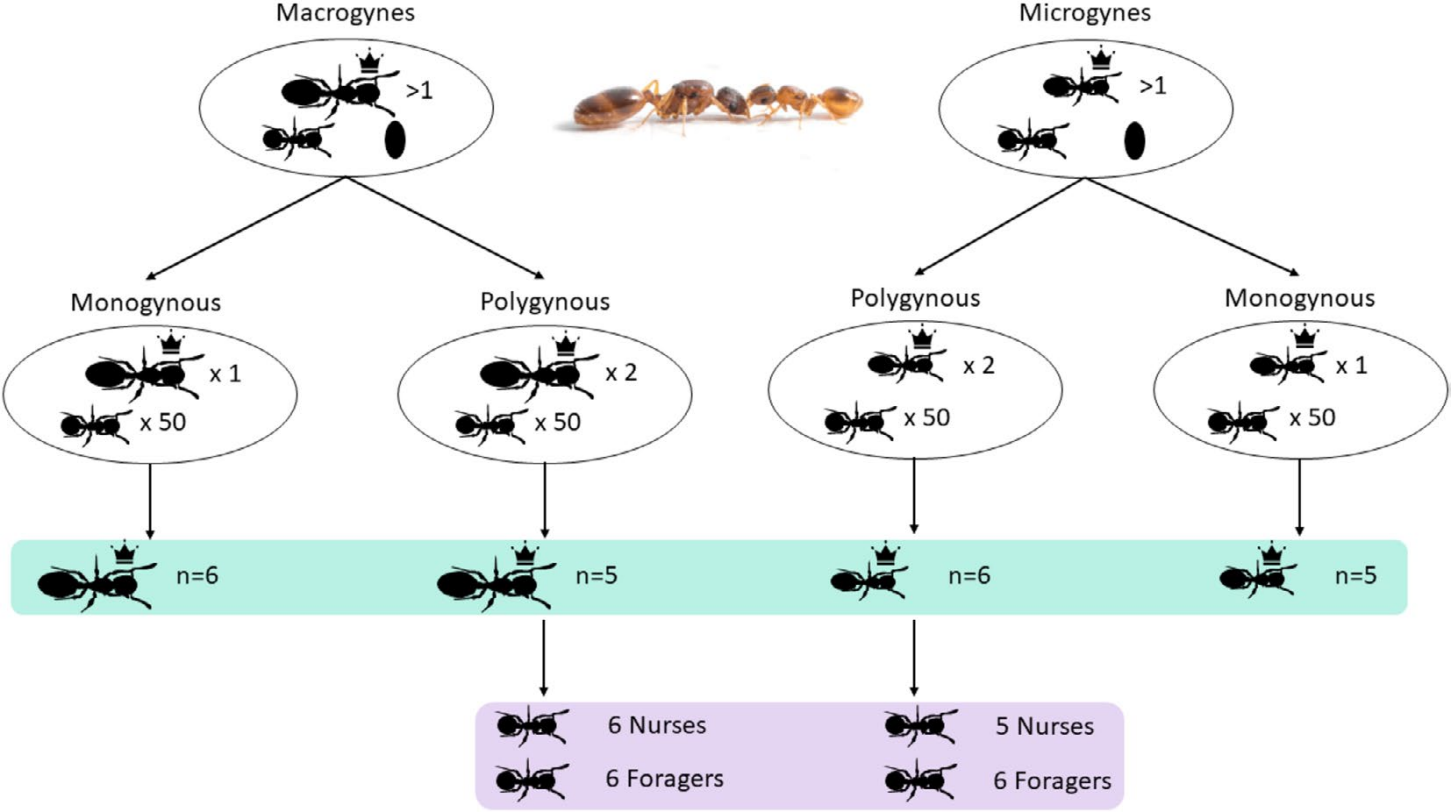
Experimental design. Colonies containing multiple queens of the same queen morph were divided into a monogyne part and a polygyne part, each with 50 workers. Samples highlighted in turquoise were used for the analysis of queen brain gene expression, whereas those in purple were used for the analysis of worker gene expression. Photo credit: Romain Libbrecht.

We used a draft genome of *T. rugatulus* (Jongepier et al. 2022) as a reference to map the reads using HISAT2 Version 2.1.0 (Kim, Langmead, and Salzberg 2015), together with Samtools Version 1.9 (Li et al. 2009) as input for StringTie Version 1.3.6 (Pertea et al. 2015) to create a genome‐guided transcriptome assembly. Alignment rates revealed one clear outlier that was removed from further analyses (Sample 562.MicMo.Q.Br; Table S3). Final replicate numbers are given in Figure 1. The quality of the resulting reference transcriptome was assessed using gffread Version 0.11.4 and TransRate (Smith‐Unna et al. 2016). Out of the 74,227 total transcripts, 41,709 had an open reading frame of at least 150 bp and were used for all following analyses. The number of coding sequences is typically much higher than the number of genes in a genome, as a single gene can produce different transcripts. BlastX Version 2.9.0 (Altschul et al. 1990) was used to annotate the transcripts with the invertebrate protein database (March 2019) with an E value threshold of 10^−5^. To obtain information on Gene Ontology terms (Ashburner et al. 2000) and KEGG pathways (Ogata et al. 1999), we ran InterProScan Version 5.36‐75.0 (Jones et al. 2014) on the translated peptide sequences (TransDecoder Version 5.5.0) of the filtered transcriptome.

The final GTF files produced by StringTie were used to obtain gene read counts using the *prepDE.py* script provided alongside the StringTie installation. We conducted two independent analyses on (a) the queen samples and (b) the worker samples (Tables S4 and S5). For analysis of the queen samples, we filtered for transcripts with read counts of at least 10 in at least four samples to obtain more reliable differentially expressed genes (DEGs), leaving a total of 28,876 transcripts for further analysis. DESeq2 Version 1.24.0 (Love, Anders, and Huber 2014) was used for gene expression analysis by performing a likelihood ratio test (LRT) comparing a full to a reduced model (Table S6 for information on models and numbers of DEGs). We assessed the effect of the following parameters on gene expression: (a) queen morph; (b) social environment; (c) the interaction of both factors. For the worker dataset, which after filtering consisted of 29,631 transcripts, we followed a similar procedure by analysing genes affected by: (a) queen morph; (b) behavioural task; (c) the interaction of both factors (Table S6 lists all models used). For both analyses, genes were considered significantly differentially expressed if FDR < 0.05 and used for further analyses. We were unable to include colony ID as a factor in our DESeq2 design, as our data were not balanced across colony identity and queen morph. To illustrate the direction of expression of the DEGs, we used DEGreport Version 1.20.0 (Pantano 2017) to generate clusters of genes with similar expression patterns. This was especially important for genes whose expression was better explained by a model incorporating the interaction of two factors, as, in those cases, the log fold change provided by DESeq2 only contains information about a single comparison of factors, while the *p* value represents the test of all variables and their interactions (see DESeq2 documentation, https://bioconductor.org/packages/release/bioc/vignettes/DESeq2/inst/doc/DESeq2.html). This clustering approach has been used before when studying the effect of multiple factors on transcriptional activity (Stoldt et al. 2023; Cheng et al. 2024; Goldberg et al. 2024). Clustering with the degPatterns function of the DEGreport package first calculates a *z*‐score, which is the mean value of expression for each gene in samples that have the same ‘col’ and ‘time’ value. In the queen dataset, we chose the queen morph as the ‘time’ parameter and the social form as the ‘col’ parameter. This means that the expression was averaged over all samples that were macrogyne‐monogyne, macrogyne‐polygyne, microgyne‐monogyne and microgyne‐polygyne. The pairwise correlation between genes is then calculated using the Kendall rank coefficient, and the clustering of genes is based on the resulting distance matrix. This approach allows us to obtain clusters of genes that change similarly in the groups of interest, regardless of the magnitude of these expression changes.

To obtain additional information beyond the gene name and functional annotation, we used the gene names to retrieve functional information from UniProt for all DEGs from the model organisms *Apis mellifera, Drosophila melanogaster* and *Caenorhabditis elegans*. The comprehensive UniProt information was then screened specifically for genes related to lifespan/longevity, fertility/fecundity and stress (Table 1), using a script available from Negroni et al. (2021). All analyses were performed in R Version 4.1.0 (Core Development Team 2020).

**TABLE 1:** List of candidate genes identified among differentially expressed genes in queens and workers using our text‐mining approach.

Gene ID	Caste	Cluster	High expression in	Adjusted *p*	BLAST hit in invertebrate database	Function in *Apis, Drosophila* or *Caenorhabditis*	References
MSTRG.6350.1	Queens	Interaction 1	Macro‐mono Micro‐poly	0.046	Zinc finger protein 2‐like isoform X3 *Temnothorax curvispinosus*	Lifespan, stress	Tehrani et al. (2014) and Singh et al. (2016)
MSTRG.23160.2	Queens	Interaction 1	Macro‐mono Micro‐poly	0.046	jmjC domain‐containing protein 5 *T. curvispinosus*	Lifespan	Ni et al. (2012)
MSTRG.21619.1	Queens	Interaction 2	Macro‐poly Micro‐mono	0.046	ATP synthase subunit b, mitochondrial isoform X1 *T. curvispinosus*	Lifespan, stress	Sun et al. (2014)
MSTRG.11670.1	Queens	Interaction 2	Macro‐poly Micro‐mono	0.046	FMRFamide receptor‐like isoform X1 *T. curvispinosus*	Stress	Iannacone et al. (2017)
MSTRG.6982.1	Queens	Interaction 2	Macro‐poly Micro‐mono	0.048	Peptidyl‐prolyl *cis*–*trans* isomerase 5, *T. curvispinosus*	Stress	GO:0070059
MSTRG.14197.1	Queens	Interaction 2	Macro‐poly Micro‐mono	0.003	Fatty acid‐binding protein, muscle isoform X2, *T. curvispinosus*	Lifespan	Ramachandran et al. (2019)
MSTRG.2959.1	Queens	Interaction 2	Macro‐poly Micro‐mono	0.046	60S ribosomal protein L9 *Megachile rotundata*	Lifespan	GO:0008340
MSTRG.10980.1	Queens	Interaction 2	Macro‐poly Micro‐mono	0.004	Superoxide dismutase [Cu‐Zn]‐like *T. curvispinosus*	Lifespan, stress	Ruan and Wu (2008)
MSTRG.6184.1	Queens	Interaction 2	Macro‐poly Micro‐mono	0.003	NADH dehydrogenase [ubiquinone] 1 beta subcomplex subunit 5, mitochondrial *T. curvispinosus*	Fecundity	GO:0000003
MSTRG.7892.6	Queens	Interaction 3	Macro‐mono Micro‐mono Micro‐poly	0.032	Zinc finger RNA‐binding protein isoform X1 *T. curvispinosus*	Fecundity	Detwiler et al. (2001)
MSTRG.21766.1	Queens	Interaction 4	Macro‐poly Micro‐mono	0.013	Superoxide dismutase [Cu‐Zn] *T. curvispinosus*	Lifespan, stress	Ruan and Wu (2008)
MSTRG.4840.1	Queens	Queen Morph 1	Micro	< 0.001	cGMP‐dependent protein kinase, isozyme 1 isoform X2 *Solenopsis invicta*	Lifespan	Hirose et al. (2003)
MSTRG.4839.1	Queens	Queen Morph 1	Micro	0.001	ATP‐binding cassette sub‐family C member Sur‐like *T. curvispinosus*	Stress	Akasaka et al. 2006
MSTRG.22936.4	Queens	Queen Morph 2	Macro	0.048	N‐terminal kinase‐like protein isoform X1 *T. curvispinosus*	Lifespan, stress	Mizuno et al. (2004) and Yan et al. (2017)
MSTRG.2449.1	Queens	Queen Morph 2	Macro	0.004	Peroxisome assembly factor 2 *T. curvispinosus*	Stress	Huang et al. (2019)
MSTRG.16927.7	Queens	Queen Morph 2	Macro	0.049	Separin *T. curvispinosus*	Fecundity	Siomos et al. (2001)
MSTRG.12340.2	Workers	Interaction 5	Macro‐brood carer Macro‐forager Micro‐brood carer	0.031	Piezo‐type mechanosensitive ion channel component 2 isoform X4 *Vollenhovia emeryi*	Fecundity	Bai et al. (2020)
MSTRG.476.3	Workers	Interaction 5	Macro‐brood carer Macro‐forager Micro‐brood carer	0.043	Kinesin‐like protein unc‐104 isoform X15 *T. curvispinosus*	Fecundity	GO:0000003
MSTRG.7288.12	Workers	Interaction 5	Macro‐brood carer Macro‐forager Micro‐brood carer	0.036	Triosephosphate isomerase isoform X1 *T. curvispinosus*	Lifespan	Gnerer, Kreber, and Ganetzky (2006) and Roland et al. (2013)
MSTRG.144.15	Workers	Interaction 5	Macro‐bd carer Macro‐forager Micro‐brood carer	0.034	5′AMP–activated protein kinase catalytic subunit alpha‐2 isoform X4 *Pseudomyrmex gracilis*	Lifespan, stress	Apfeld et al. (2004) and Lee et al. (2008)
MSTRG.4441.1	Workers	Interaction 8	Macro‐forager Micro‐brood carer	0.005	Major royal jelly protein 1‐like *T. curvispinosus*	Fecundity	Kamakura (2011)

***Note:*** For each gene, available references were retrieved from the functional information in UniProt when possible. If UniProt references were unavailable, a literature search was conducted using the three model organisms (*Apis mellifera, Drosophila melanogaster* and *Caenorhabditis elegans*). In cases where no references were found, the Gene Ontology (GO) ID from UniProt, which led to the identification of the gene, is provided.

GO enrichment analyses on the lists of DEGs belonging to the different groups were performed using topGO Version 2.36.0 (Alexa and Rahnenfuhrer 2018). We used the weight01 algorithm and the Fisher's exact test implemented in the package to test for overrepresentation of functional terms in our gene lists of interest. As different genes are expressed in queens and workers, we used a caste‐specific gene set as the universe, that is, as the reference data set, against which the test set, that is, the DEGs, was tested.

## 3 Results

### 3.1 Influence of Queen Morph and Social Environment on Brain Gene Expression in Queens

**FIGURE 2:**
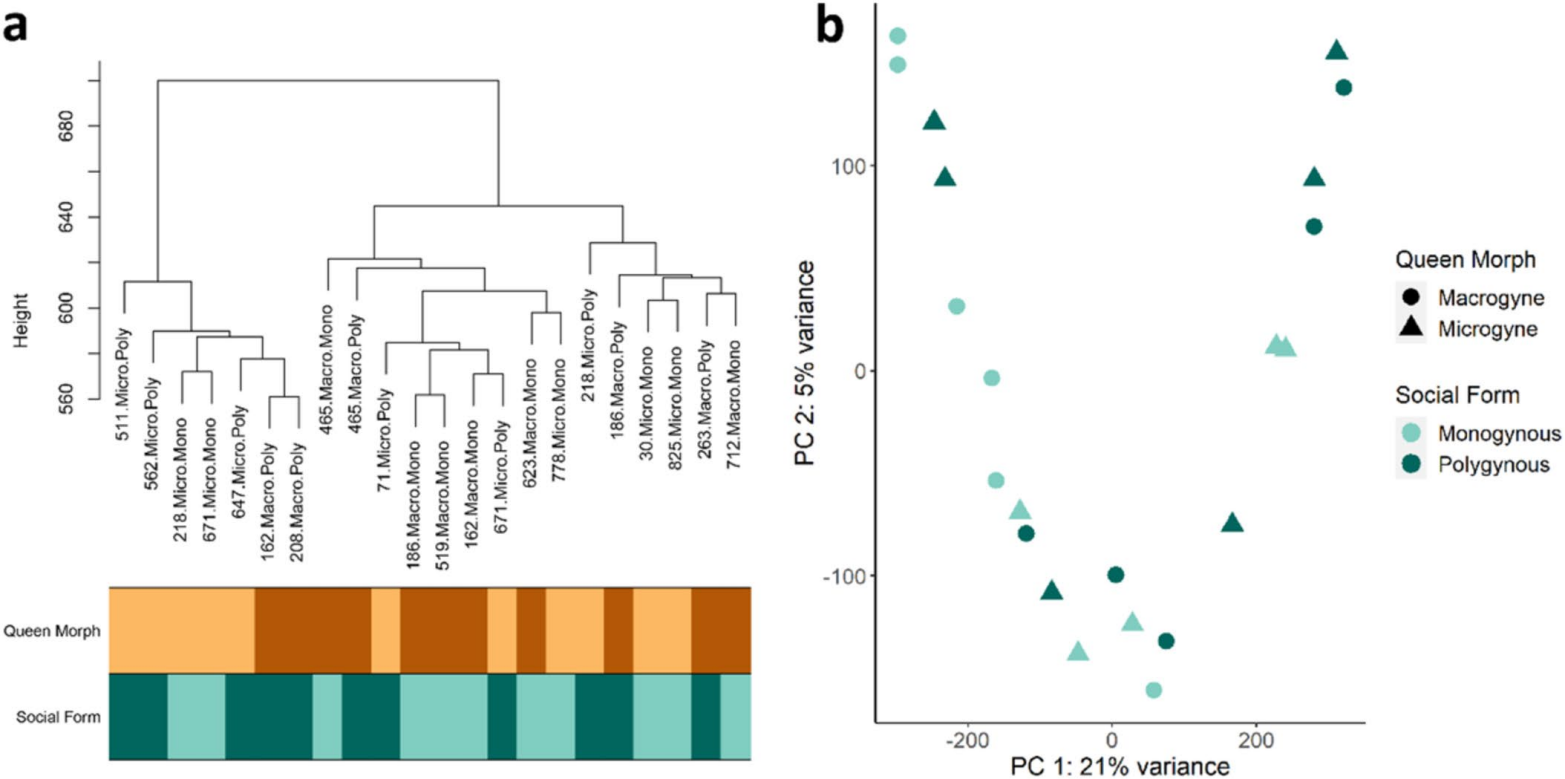
Clustering of queen samples according to social environment and queen morph based on the filtered transcript count matrix for queens. (a) Sample dendrogram created using hierarchical clustering. Labels indicate sample names with, the first 3‐digit number corresponding to the source colony, the next part corresponding to the queen morphs with ‘Macro’ for macrogyne queens and ‘Micro’ for microgyne queens. The last part denotes the social environment treatment with ‘Mono’ for monogynous colonies and ‘Poly’ for polygynous colonies. (b) Principal component analysis based on all transcripts. Samples are coloured according to the social environment, and the shapes represent the respective queen morph.

Queen samples clustered neither clearly by queen morph nor by social environment (Figure 2a,b). Also, colony ID did not affect sample grouping (Figure S1), which, in addition to the inhomogeneity of sample origin, led us to exclude colony identity as a factor in the differential expression analysis. We detected 39 genes affected in their expression by queen morph, which could be grouped into two clusters. Cluster Q2 contained 27 DEGs showing higher expression in macro‐ versus microgynes, and Cluster Q1 contained 12 DEGs showing the reversed pattern (Figure S2a). Genes in Cluster Q2 were enriched for DNA integration. Social environment was linked to the expression of 15 genes; one cluster contained eight genes that were higher expressed in monogyne queens (Cluster Q4), while the other seven genes showed stronger expression in polygynous queens (Cluster Q3; Figure S2b). Both clusters showed no functional enrichment.

The interaction between queen morph and social environment affected the highest number of genes, namely 63, which could be grouped into four clusters (Figure 4a). The largest Cluster, Q5, comprised 31 genes exhibiting high expression in macrogynous queens residing in monogynous colony and a low expression for poly‐macrogyne queens, with a reversed pattern in colonies with microgyne queens. Cluster Q6, including, 22 DEGs exhibited the reversed pattern. Cluster Q8, comprising only eight genes, showed an expression similar to Cluster Q6, with expression being high for macrogynes in polygyne colonies and microgynes in monogyne ones. Functional enrichment of genes in clusters was only detected for Q6, which was enriched for ‘isoprenoid biosynthetic process’.

To obtain more specific functional information for the candidates from our DEG set, we used their best BLAST hits as search queries in UniProt for three model organisms: *A. mellifera, D. melanogaster* and *C. elegans*. Using a text‐mining approach based on reviewed UniProt annotations, we were able to identify nine genes involved in the regulation of lifespan (see Table 1), two genes directly related to fecundity and nine candidates involved in stress by parsing the annotated lists of DEGs for the terms ‘lifespan’ and ‘longevity’, ‘fecund’, ‘fertil’ and ‘reproduct’ and ‘stress’. Cluster Q6, containing genes influenced by the interaction of queen morph and social environment, showed a high number of genes related to both lifespan and stress, including a gene encoding a superoxide dismutase similar to a gene found in Cluster Q8 (Figure 5a).

### 3.2 Influence of Queen Morph and Behavioural Type on Brain Gene Expression in Workers

**FIGURE 3:**
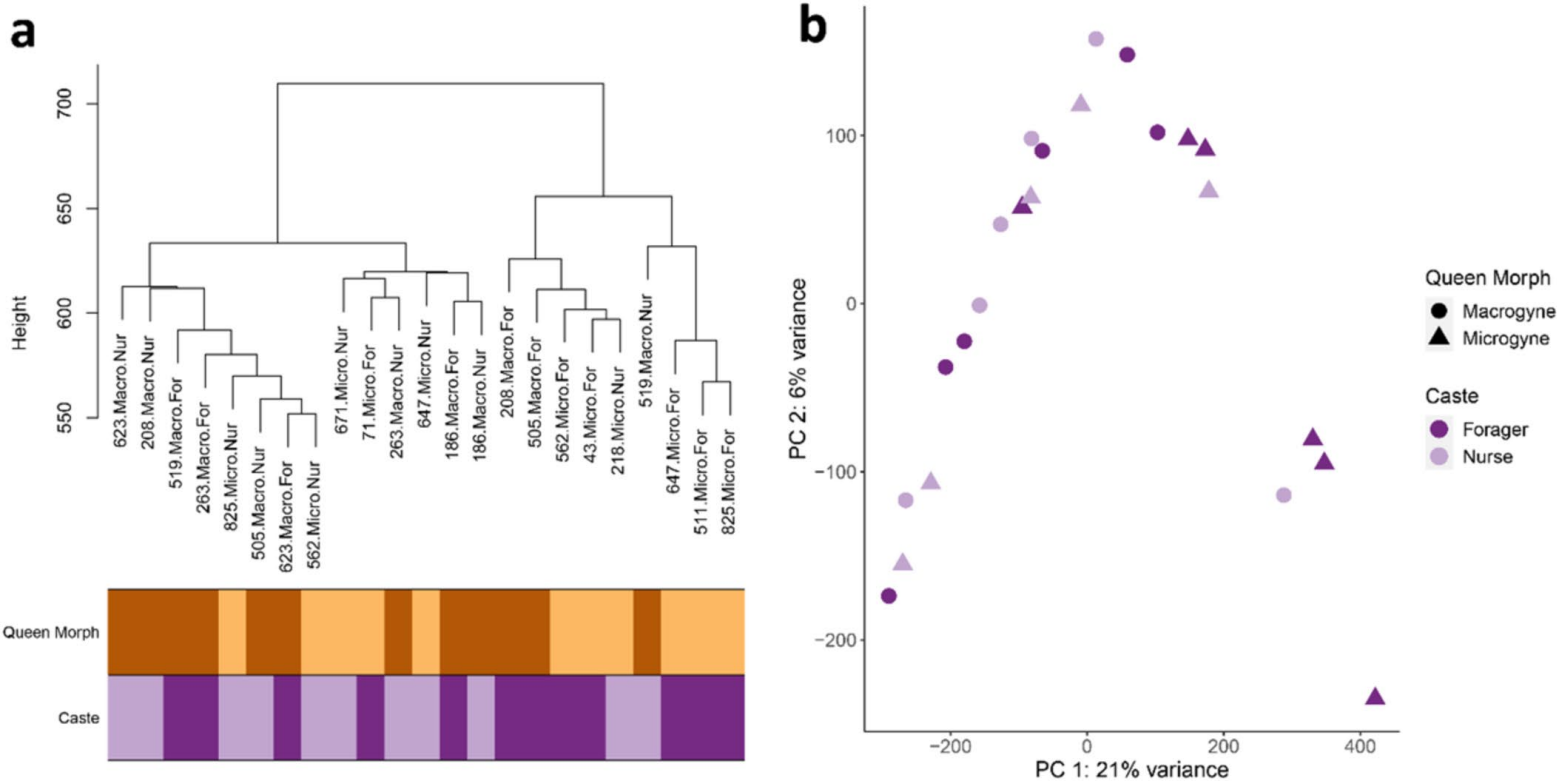
Clustering of worker samples according to behavioural type and queen morph based on the filtered transcript count matrix for workers. (a) Sample dendrogram created using hierarchical clustering. Labels again indicate the following meta‐data: The first part corresponds to the source colony; the next part corresponds to the queen morph, with ‘Macro’ for the residing queens being macrogynes and ‘Micro’ for microgyne queens. The last part represents the behavioural type of the worker samples, with ‘For’ standing for foragers and ‘Nur’ for nurses. (b) Principal component analysis based on all transcripts. Samples are coloured according to the behavioural type, and the shapes represent the respective queen morph of the residing queens.

**FIGURE 4:**
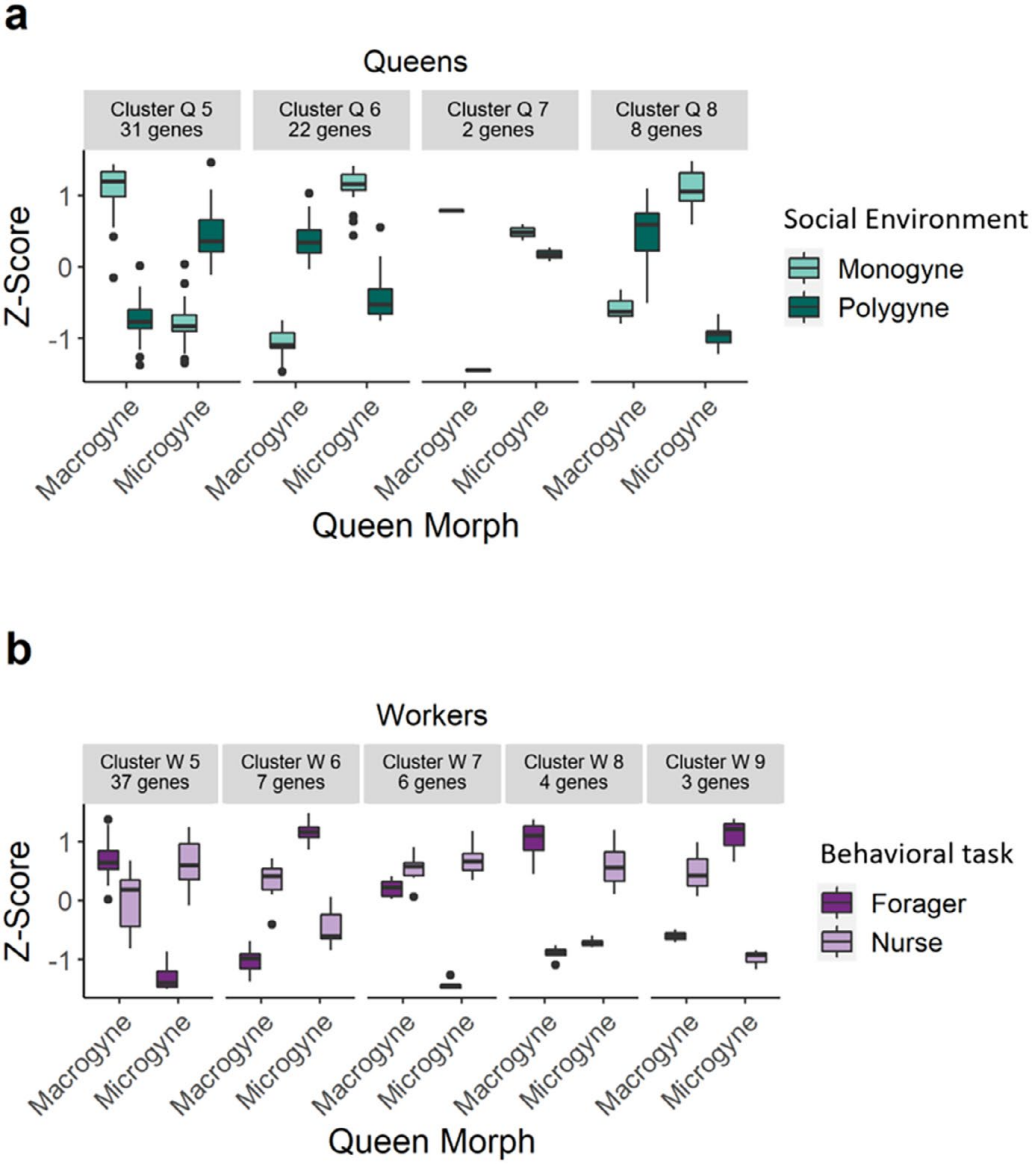
Clustering of differentially expressed genes using DEGreport for: (a) DEGs influenced by the interaction of queen morph and social environment in queens and (b) DEGs influenced by the interaction of behavioural task and queen morph in workers.

The worker samples clustered neither according to queen morph, behavioural task nor colony identity in the sample dendrogram nor in the PCA, including all transcripts (Figure 3). The results of our expression analysis on the brains of workers showed that only two genes were differentially expressed according to behavioural tasks, 14 genes were affected by queen morph and again the majority was influenced by the interaction, this time between queen morph and behavioural type, with a total 57 genes. Genes differentially expressed according to queen morph are clustered into two clusters: one with higher expression in macrogynes containing 11 genes (Cluster W2) and one with higher expression in microgynes and containing three genes (Cluster W1; Figure S3a). Behaviour‐specific genes resulted in two clusters, consisting of one gene each with higher expression in nurses (Clusters W3 and W4). The transcripts affected by the interaction are grouped into five clusters (Figure 4b). The largest Cluster, W5, comprised 37 genes with low expression in foragers of microgyne colonies and was functionally enriched for ‘meiotic cell cycle’, though this was based on a single gene that, according to our BLAST, encoded an uncharacterised protein LOC112452299 in *Temnothorax curvispinosus*. We manually blasted the nucleotide sequence of this gene against the complete nr database using the BlastX algorithm, which revealed that the next closest hit with a functional annotation was a MEIOC protein in *Acromyrmex charruanus* (query cover: 76%; identity: 92.89%; *E* value: 0.0). The MEIOC protein is known to extend the meiotic phase in mice, allowing for completion of this reproductive event (Abby et al. 2016; Soh et al. 2017).

**FIGURE 5:**
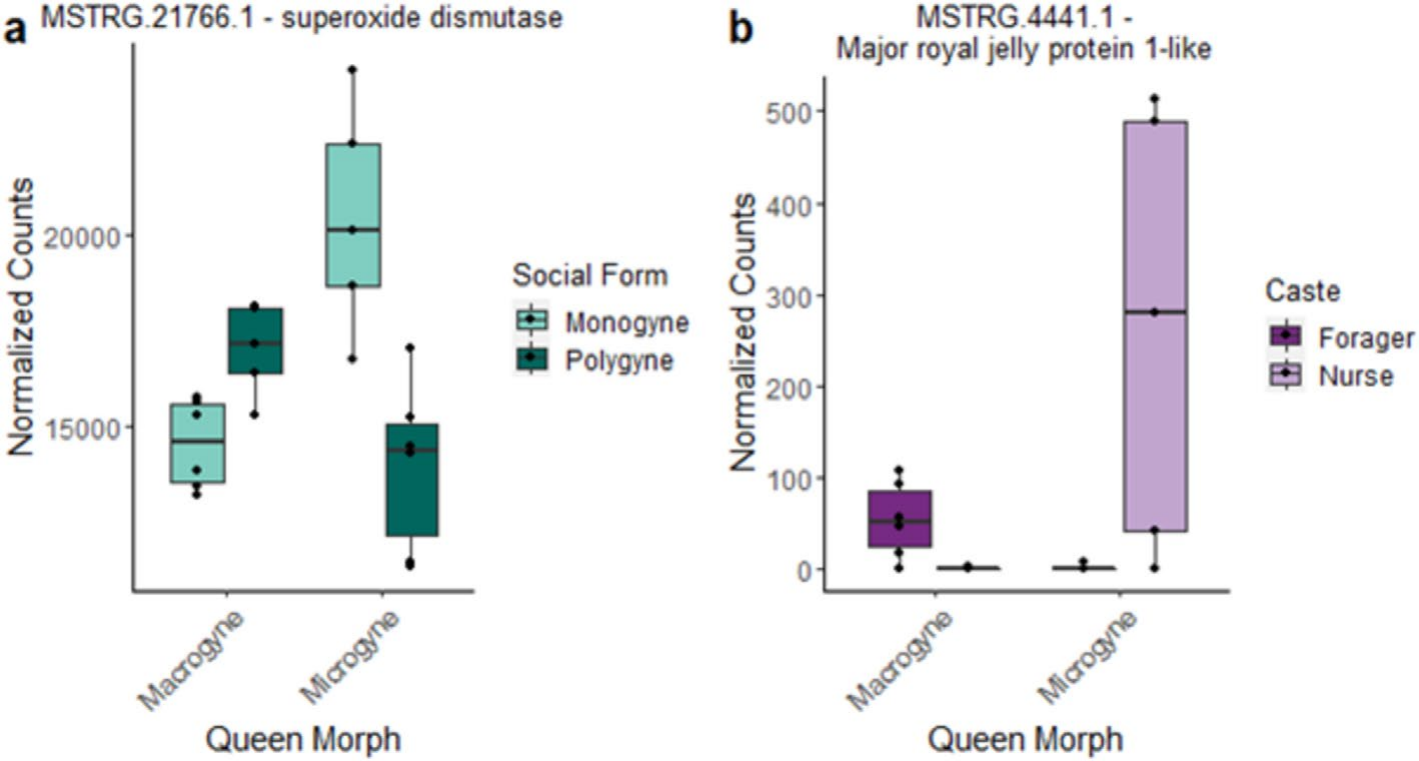
Expression of candidate genes in brains of (a) queens and (b) workers.

Our text mining approach revealed three genes linked to fecundity, two to longevity and one to stress, the last one overlapping with the ones involved in longevity (Table 1). The only hit we found for *A. mellifera* among all differentially expressed transcripts was one encoding major royal jelly protein 1‐like, a protein known to be involved in caste determination and queen reproduction (Kamakura 2011). This gene was found in Cluster W8 of the transcripts influenced by an interaction of queen morph and behavioural type, and was lowly expressed in foragers from colonies with a microgyne queen but high in all other groups (Figure 5b).

## 4 Discussion

The ant *T. rugatulus* exhibits a queen polymorphism, which is loosely associated with the social environment of the colony, namely the number of resident queens. Using a long‐term experimental manipulation of ant colonies in a full‐factorial design, our well‐replicated study disentangled the influence of queen morph and social environment on the transcriptional activity in the brains of *T. rugatulus* queens. Moreover, we analysed worker transcriptomes to investigate the effect of queen morph and behavioural task. Our expression analyses revealed that transcriptional activity in the brains of queens and workers predominantly depends on interactions between queen morph and either social environment or behavioural task. By investigating the influence of the two factors and their interactions, we were able to unravel patterns that would have been masked in single‐factor analyses. The transcriptional activity of many genes showed an inverse response to queen number in macrogynes as opposed to microgynes, indicating that these two queen morphs differently adjust to their social environment. Similarly, in the workers, the activity of the largest number of genes was not dependent on their behavioural task or social environment as such, but on the interaction of the two factors. This means that there were many genes whose expression showed an inverse pattern in nurses and foragers from macrogyne or microgyne colonies.

### 4.1 Queen Morphs Differ in Their Response to Changes in the Social Environment

We experimentally disentangled queen morph from the social environment to study the influence of both factors on transcriptional activity in the brain and to explore the interaction between the two factors, which proved to be particularly strong. In the invasive fire ant *S. invicta*, social environment and queen form are closely linked, and the expression of hundreds of genes differs between SB/SB queens from monogynous colonies and SB/Sb queens from polygynous colonies (Martinez‐Ruiz et al. 2020). Transcriptional activity in the brain of young fire ant queens embarking on the nuptial flight depended on both the social environment in the mother colony and the queen's genotype on the social chromosome (Arsenault et al. 2020). This study also uncovered supergene‐associated gene upregulation, allele‐specific expression and pronounced extra‐supergene trans regulatory effects. Our finding of a weak main effect of queen morph on transcriptional activity in the brain may also be due to the focal tissue, as *T. rugatulus* queens of the two morphs differ strongly in the gene expression of the fat body, especially in the expression of metabolic genes (Negroni et al. 2021). Thus, in our analyses we expected to find strong expression differences between queen morphs and possibly between social environments. However, thanks to our full factorial design, we were able to investigate the interaction between these two factors. Indeed, our analysis revealed that the majority of DEGs was influenced by an interaction of both factors (63 in total). Half of these transcripts (*N* = 31) were upregulated in macrogyne queens in monogynous societies in contrast to microgyne queens in polygynous colonies. This expression pattern corresponds to the social structure in which both queen morphs are typically found. Additionally, two other clusters (Q6 and Q8) showed the reversed pattern, with high expression in queens in their atypical environment. This indicates that the two queen morphs respond to their (un)usual environment via the expression of a similar but small set of genes. We propose social stress in an unaccustomed social environment as the most likely explanation for this finding. If the two morphs exhibit adaptations to their respective social environments, our experimental design might have induced stress in queens when placed in a social environment they are not accustomed to. A form of social stress has already been reported in harvester ants, where queens that usually found colonies on their own showed a higher number of aggressive events when kept with other queens (Clark and Fewell 2014). But why would microgynes experience social stress when kept alone? Negroni et al. (2021) previously reported that while the number of eggs between mono‐ and polygynous colonies did not differ, but the egg‐laying rate of individual queens was reduced in polygynous colonies. Thus, microgyne queens, which are accustomed to sharing the task of reproduction with other queens, could experience social stress when kept alone, as they need to increase their egg‐laying rate. Our hypothesis is further supported by the candidate genes found among the DEGs influenced by the interaction: Among those we also detected some related to lifespan and stress, including *superoxide dismutase* (Beckman and Ames 1998; Shen et al. 2013). Indeed, s*uperoxide dismutase* was found to be upregulated in the brain and fat body of older macrogynes compared to young founding queens (Negroni, Foitzik, and Feldmeyer 2019).

Among the 39 DEGs between the two morphs, independent of social structure, we identified two candidate genes encoding two kinases, of which one was more highly expressed in macrogynes and the other in microgynes. The latter is a cGMP–dependent protein kinase that may not only be involved in regulating lifespan by interfering with the insulin pathway in *C. elegans* (Hirose et al. 2003), but also plays a role in the adaptation to odours (Levy and Bargmann 2020). The other gene of interest was annotated as Gp‐9, which encodes a putative odorant‐binding protein in the invasive fire ant (*S. invicta*) (Krieger and Ross 2002; Ross 1997). This gene is in linkage disequilibrium with a supergene containing approximately 500 genes that determine the social morph of queens (Gotzek and Ross 2009; Lucas, Nicolas, and Keller 2015; Pracana et al. 2017; Chiu et al. 2020). The upregulation of this gene in macrogynes of *T. rugatulus* may indicate a higher sensitivity to some nestmate‐specific odours compared to ants from the microgynous social form. Further ongoing studies of the genomes and their organisation in the two queen morphs will elucidate the existence and composition of potential social chromosomes in this species.

### 4.2 Queen Morph Influences Transcriptional Activity in Worker Brains

In *S. invicta* and *T. rugatulus* queen morph also influences worker traits such as size (Goodisman et al. 1999; Choppin et al. 2021). Hence, we were interested in seeing whether queen morph affects brain gene expression in workers differentially, with those mainly working in the colony as nurses compared to those mainly active outside as foragers. Our expectation was that transcriptional activity in worker brains would vary mainly between behavioural tasks, that is, whether a worker is a forager or nurse, as task was shown to influence transcriptional activity in workers more than age and fertility (Kohlmeier et al. 2019). Next, we assumed that queen morph might influence worker gene expression, as previous studies showed differences in colony dynamics between the morphs (Choppin et al. 2021). For example, microgyne queens are fed more often by workers, which might trigger the expression of metabolism‐ and nutrition‐related genes in these workers (Negroni et al. 2021). In our study, however, brain gene expression in workers was hardly dependent on their role in the colony (2 DEGs) but varied with the queen morph (14 DEGs) and especially with the interaction of task and morph (57 DEGs). Again, our results thus indicate that microgyne and macrogyne workers adjust differently to their behavioural task in the colony. Our findings contrast with an analysis of direct and indirect genetic effects of the *S. invicta* supergene on gene expression in the brain and abdomen of workers and gynes (Arsenault et al. 2023), which revealed a strong direct influence on transcriptional activity across different tissues and castes. In contrast, indirect genetic effects were only detected when the genotype of the social chromosome of the social partners differed throughout the development and adult life of the focal workers.

When focusing on the genes and their functions influenced by the interaction between behavioural type and social form, we identified a gene in a cluster highly expressed in macrogyne‐foragers and microgyne‐nurses (W8), which encodes a major royal jelly protein 1‐like. In honeybees, nine major royal proteins exist, the one found in this study, major royal jelly protein 1, is encoded by the *mrjp1* gene (Buttstedt, Moritz, and Erler 2014). This gene was previously found to be higher expressed in the head of forager bees compared to caged workers but no different to nurses (Buttstedt, Moritz, and Erler 2013), and is suggested to be important for nutrition and social behaviour in honeybees (Schmitzová et al. 1998; Drapeau et al. 2006). In honeybee nurses, royal jelly is produced to feed the larvae and to determine their caste. Interestingly, vitellogenin, a protein also important for caste determination in ants (Kohlmeier, Feldmeyer, and Foitzik 2018), was shown to be source of the royal jelly produced by workers (Amdam et al. 2003). The high expression in nurses in microgynous colonies could therefore actually be explained by their higher trophallactic activity towards the queens (Negroni et al. 2021). Additionally, we were able to detect some candidate transcripts related to longevity, fecundity and stress, including *superoxide dismutase*, but further functional studies, for example, using RNAi knockdown, would be necessary to evaluate their role in shaping worker life‐history in relation to the colony queen morph.

### 4.3 Conclusions

In this study, we experimentally altered the social environment of an ant species with two queen morphs, each with divergent reproductive strategies, to investigate the effects on gene activity in the brain. We predicted that macrogyne and microgyne queens would be adapted to head their colony alone or to reproduce together with others, respectively. In fact, our gene expression study shows that transcriptional activity in the brain depends on an interaction between queen morph and social environment. Surprisingly, we found little evidence that queen morph or social environment alone strongly affects gene expression. Rather, our results indicate that different queen phenotypes of an ant species may be closely adapted to their social niches. Moreover, our results show that shifts in molecular physiology, even if not very pronounced, can spill over to the worker caste. Differences in worker physiology have already been demonstrated in the fire ant *S. invicta* (Lucas, Nicolas, and Keller 2015), where queen morph is determined by a social chromosome (Wang et al. 2013). Our results reveal that differences in gene expression in the brain of workers may be influenced by an interaction between behavioural caste and queen form. This study thus sheds light on how phenotypes and social environment interact to influence the molecular physiology of social insects.

## Author Contributions

S.F., B.F. and M.A.N. conceived the study. The experiment and data collection was conducted by M.A.N., and the analyses by M.S. M.S. and M.A.N. wrote the first version of the manuscript. All authors improved the manuscript and its revision.

## Conflicts of Interest

The authors declare no conflicts of interest.

## Supporting information


Figures S1–S3.

Tables S1–S6.

## Data Availability

The raw reads used for this study are available on the Sequence Read Archive under Bioproject ID PRJNA608262. R scripts for analysis, gene count matrices, transcriptome sequences, Blast Hits and InterProScan output can be found on Mendeley under the DOI 10.17632/n3ctsgzdyr.1.
