# Supplementary material for: Molecular Adjustment to a Social Niche: Brain Transcriptomes Reveal Divergent Influence of Social Environment on the Two Queen Morphs of the Ant Temnothorax rugatulus
Source: Mol Ecol. 2025 Jan 7;34(15):e17649. doi: 10.1111/mec.17649 (PMC12288803; doi:10.1111/mec.17649)
Supplement: Supplementary file 1 — Figures S1–S3. [file MEC-34-e17649-s002.pdf]

**Supplementary Figures**

# Molecular adjustment to a social niche: Brain transcriptomes reveal divergent influence of social environment on the two queen morphs of the ant *Temnothorax rugatulus*

Marah Stoldt[1], Matteo Antoine Negroni[1,2], Barbara Feldmeyer[3], Susanne Foitzik[1]

[1]*Institute of Organismic and Molecular Evolution, Johannes Gutenberg University Mainz, Germany*

[2]*Department of Biology, University of Fribourg, Chemin du Musée 10, 1700 Fribourg, Switzerland*

[3]*Senckenberg Biodiversity and Climate Research Center (SBiK-F), Molecular Ecology, Frankfurt, Germany*

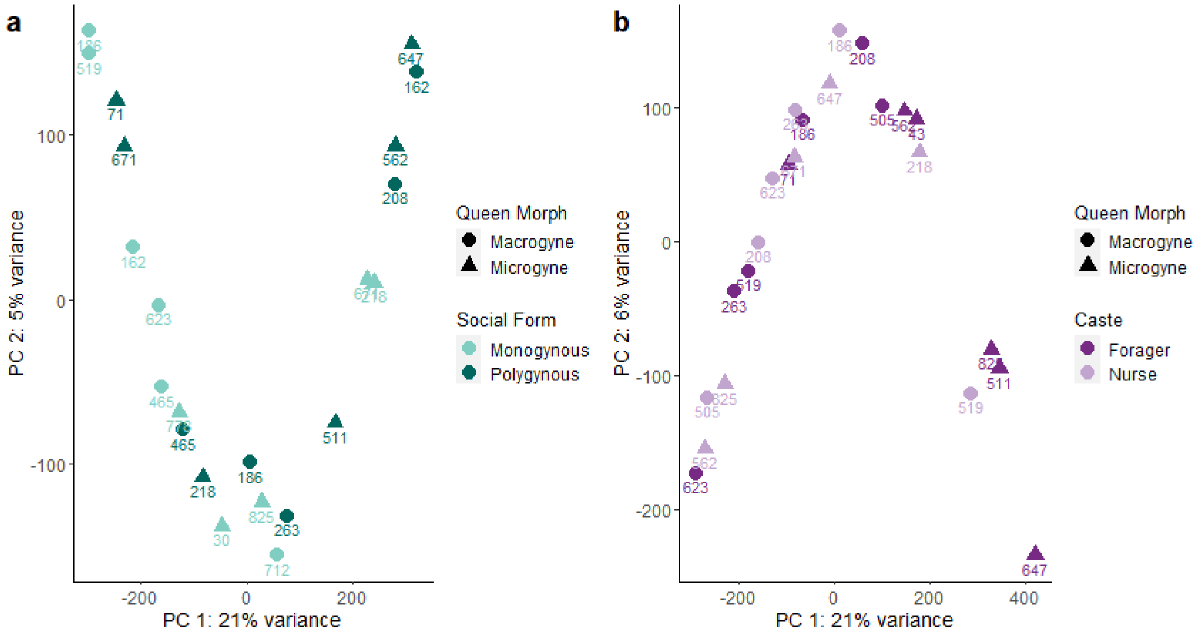


**Figure S1** PCAs on brain transcriptome data including all expressed genes with labels corresponding to source colony ID for **a.** queens and **b.** for workers.

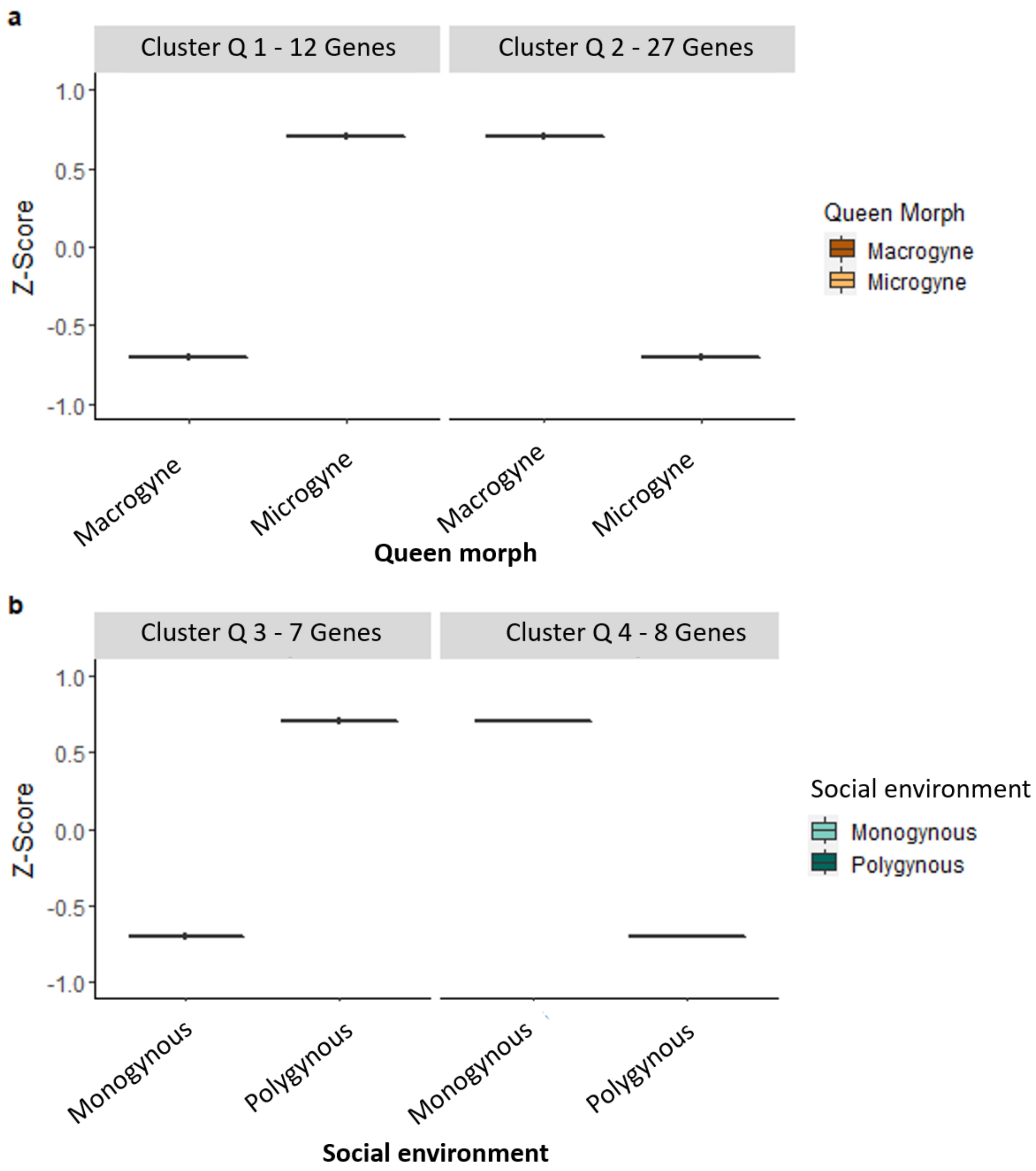


**Figure S2** Clustering of differentially expressed genes in queens using DEGreport for **a.** DEGs influenced by queen morph **b.** DEGs influenced by social environment.

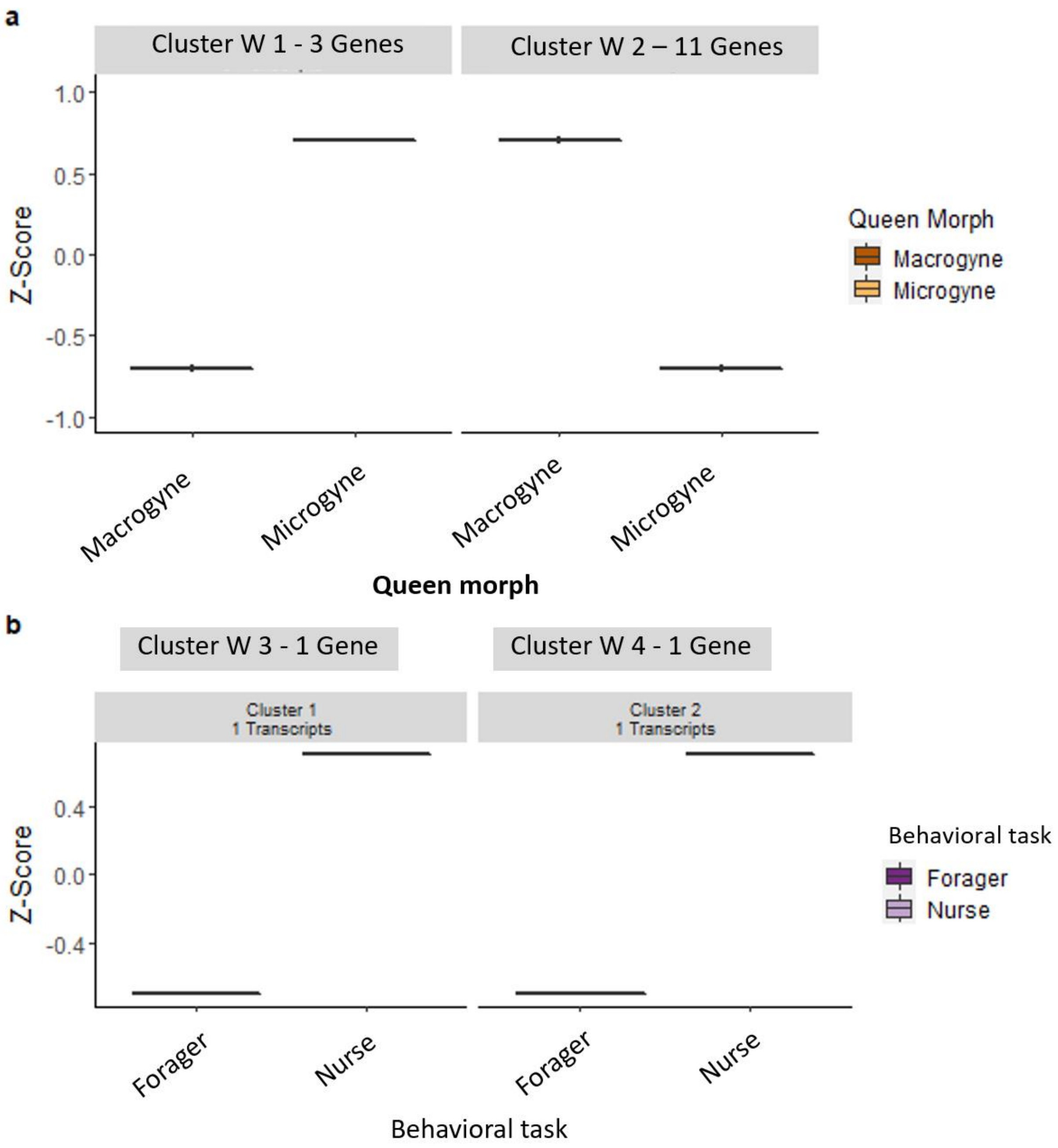


**Figure S3** Clustering of differentially expressed transcripts in workers using DEGreport for **a**. DETs influenced by queen morph **b.** DETs influenced by behavioral type.
